# Longitudinal serological detection of exposure to SARS-CoV-2 in a cohort of pregnant women in Malawi: a secondary analysis from a randomised controlled trial

**DOI:** 10.1136/bmjopen-2025-113123

**Published:** 2026-06-29

**Authors:** Louise M Randall, Nicholas Kiernan-Walker, Ernest Moya, Glory Mzembe, Alistair R D McLean, Rebecca Harding, Ramin Mazhari, Gomezgani Mhango, Katherine L Fielding, Ivo Mueller, Martin N Mwangi, Sabine Braat, Kamija Phiri, Sant-Rayn Pasricha, Emily M Eriksson, Ricardo Ataíde

**Affiliations:** 1 Infection and Global Health, The Walter and Eliza Hall Institute of Medical Research, Melbourne, Victoria, Australia; 2 The Training and Research Unit of Excellence, Blantyre, Malawi; 3 Methods and Implementation Support for Clinical and Health Sciences Research Hub, The University of Melbourne, Melbourne, Victoria, Australia; 4 Department of Medical Biology, The University of Melbourne, Melbourne, Victoria, Australia; 5 Healthy Mothers Healthy Babies, Micronutrient Forum, Washington DC, North West, USA; 6 Division of Human Nutrition and Health, Wageningen University, Wageningen, Netherlands; 7 School of Population and Global Health, The University of Melbourne, Melbourne, Victoria, Australia

**Keywords:** Pregnancy, COVID-19, Public health, INFECTIOUS DISEASES

## Abstract

**Abstract:**

**Objective:**

To investigate whether IgG to severe acute respiratory syndrome coronavirus 2 (SARS-CoV-2) antigens could reveal undetected SARS-CoV-2 exposure in a cohort of Malawian pregnant women participating in the REVAMP clinical trial.

**Design:**

A secondary analysis of serological samples from a randomised controlled trial of iron supplementation in pregnancy, which began recruiting women in November 2018 and had its last visit occurring in September 2021.

**Setting:**

Resource-limited setting in Zomba and Blantyre, Southern Malawi.

**Participants:**

Pregnant women with ultrasound-confirmed singleton pregnancies in the second trimester, with haemoglobin <100 g/L and randomised at enrolment to receive either intravenous ferric carboxymaltose or standard oral iron, where no coronavirus disease 2019 (COVID-19) clinical cases or SARS-CoV-2 positive tests were reported during the pandemic (April 2020–September 2021).

**Primary and secondary outcomes:**

Primary outcome was the levels of antibodies and seropositivity to SARS-CoV-2 antigens in the cohort of women across the duration of the trial. Secondary outcomes were the impact of IgG levels and seropositivity to SARS-CoV-2 on pregnancy outcomes.

**Results:**

At delivery, IgG levels to SARS-CoV-2 antigens increased sharply by 18.5%–29.7% every 30 days during COVID-19 waves 2 and 3. Overall seropositivity reached 39.3% during the pandemic; however, 14.7% pre-pandemic seropositivity demonstrates cross-reactive antibody responses. Pandemic pregnancies showed improved outcomes with longer gestations (mean difference: 0.6 weeks (95% CI 0.2 to 0.9)) and higher birth weights (mean difference: 169.3 g (65.9–272.6)). SARS-CoV-2 IgG levels were not associated with pregnancy outcomes.

**Conclusion:**

Serological testing was able to detect exposure to SARS-CoV-2 in a population without clinical indications of the disease, suggesting that serosurveillance is more sensitive than relying on clinical data to monitor pathogen exposure in the community. Additionally, this highlights pregnancy cohorts as valuable sentinel populations for infectious disease surveillance in resource-limited settings.

**Trial registration number:**

This trial was prospectively registered at ANZCTR: ACTRN12618001268235.

**STRENGTHS AND LIMITATIONS OF THIS STUDY**

- This study relied on a well characterised cohort of pregnant women who were part of a randomised controlled trial and were longitudinally monitored throughout pregnancy.
- The REVAMP trial data did not collect specific information regarding COVID-19 vaccination status of the participants.

## Introduction

 The COVID-19 pandemic presented unique surveillance challenges in resource-limited settings, where constrained testing capacity and limited healthcare access complicated efforts to track SARS-CoV-2 transmission.^1^ These challenges were noted in Malawi and while official statistics suggested low case numbers compared with other global regions, limited molecular testing capacity and accessibility, particularly in rural areas, left the true extent of viral spread uncertain.^2 3^

This was evident in the REVAMP trial (Nov 2018–Sep 2021), a randomised controlled trial of iron supplementation in pregnancy conducted in southern Malawi,^4^ which spanned three COVID-19 waves.^5^ Despite frequent monitoring across at least four antenatal visits, no participant reported a positive test or presented with respiratory symptoms indicative of COVID-19. Yet a seroprevalence survey of Malawi Blood Transfusion Service donors indicated that the seropositivity in Malawi could reach 65%,^6^ suggesting that undetected infection within our closely monitored cohort was likely. Serological surveillance, when properly validated, overcomes detection gaps by measuring antibodies to reveal past exposure and population-level transmission patterns missed by traditional methods.^7^ The value of this approach has been well-demonstrated in various infectious disease contexts.^8^,^11^

Pregnancy cohorts are particularly valuable for serosurveillance, as they provide year-round contact points for sample collection and monitoring.^12 13^ For example, in malaria-endemic African regions, serosurveillance of pregnant women is increasingly seen as an efficient way to track malaria transmission trends.^10 12 13^ Additionally, pregnancy-specific interventions, such as vaccinations during governmental health programmes, create built-in immunological controls that can validate changes seen in incidental serological findings, for example, tetanus vaccination in pregnancy offers a reliable test of both healthcare delivery and antibody detection.^14^

The REVAMP trial presented an ideal platform for investigating potential undetected transmission of SARS-CoV-2 through serosurveillance during pregnancy. Its timeline included pre-pandemic and post-pandemic samples from the same population—rare in COVID-19 serological studies—while regular monitoring ensured consistent sample collection during the pandemic. The comprehensive data available on demographics, clinical outcomes and healthcare delivery patterns offered multiple avenues for validating and assessing evidence of undetected SARS-CoV-2 exposure. In addition, we evaluated the impact of changes to SARS-CoV-2 serology on pregnancy outcomes.

## Methods

### Study design

All samples are from the REVAMP trial, which took place in southern Malawi, in the Zomba and Blantyre districts. The trial protocol, statistical analysis plan and primary results have been published.^4 15^ Briefly, pregnant women with ultrasound-confirmed singleton second-trimester pregnancies were randomised to intravenous ferric carboxymaltose (Vifor Pharma, St. Gallen, Switzerland) or standard oral iron if eligible as determined by haemoglobin of <100 g/L(by HemoCue 301+ (Angelholm, Sweden, HemoCue AB)) and a negative malaria rapid diagnostic test among others.^4^ The primary endpoint of the REVAMP trial was anaemia prevalence at 36 weeks’ gestation. Venous blood samples used in this study were collected at enrolment (13–26 weeks’ gestation) and at delivery. Blood was processed within 2 hours of collection and stored at −80°C until shipment to Melbourne, Australia. All women were eligible for government-provided pregnancy immunisations, including tetanus toxoid (Tt) at the first antenatal visit, a second dose after 4 weeks and a third dose 6 months later.^16^

The trial received ethics approvals from the College of Medicine, University of Malawi, Malawi (P.02/18/2357) and the Walter and Eliza Hall Institute (WEHI), Australia (18/02), and was prospectively registered (ACTRN12618001268235). An independent data and safety monitoring board oversaw the trial. Secondary analyses of the trial samples were included in the study protocol.

#### COVID-19 setting

**Figure 1:**
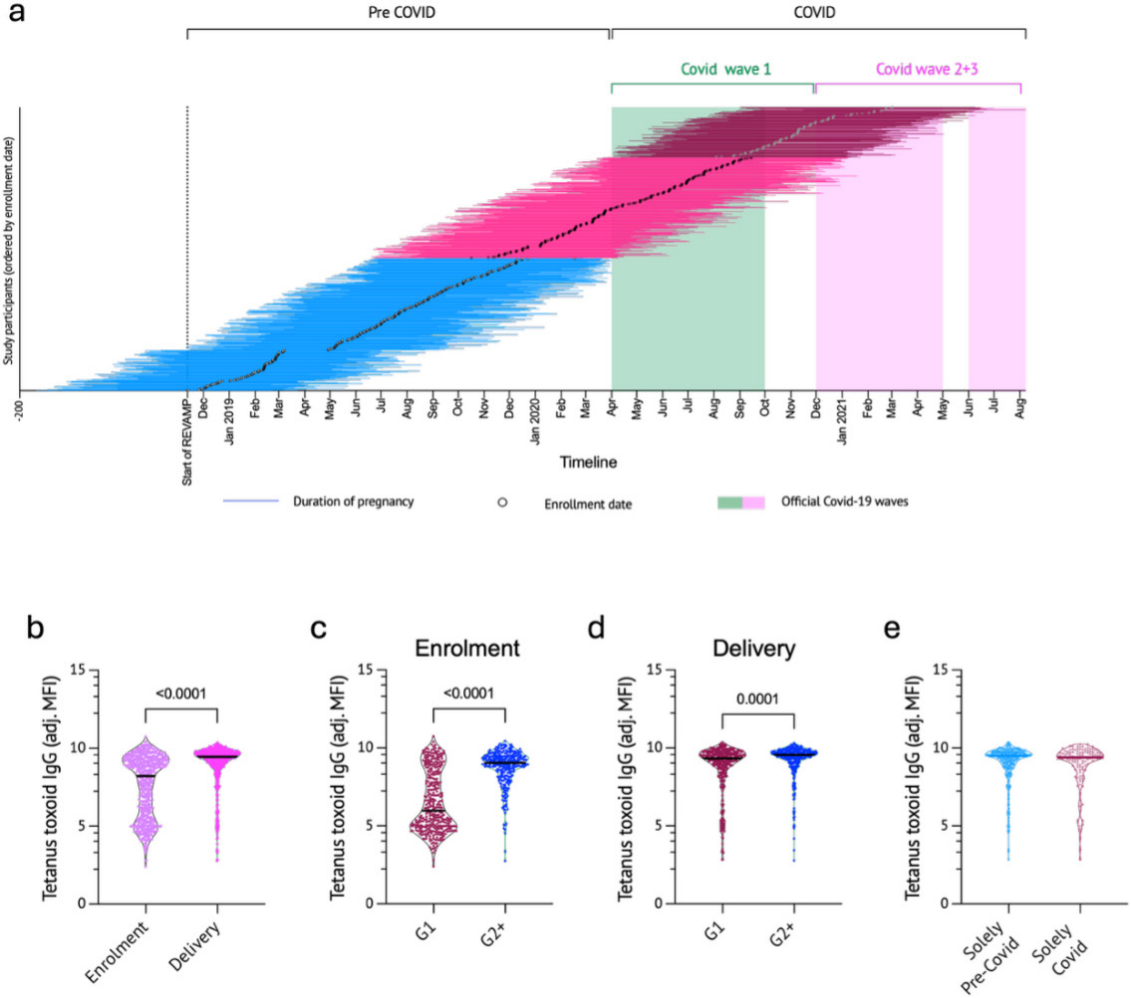
Participant timelines and cohort characteristics across pre-pandemic and pandemic periods. (**a**) Individual participant timeline distribution across the study period. Horizontal lines represent each participant’s pregnancy duration, with enrolment dates marked as circles. Blue horizontal lines are pregnancies that occurred exclusively in the pre-COVID-19 period. Magenta lines are pregnancies that occurred partially in the COVID-19 period. Dark red lines are pregnancies entirely occurring within the COVID-19 period. Shaded boxes represent reported COVID-19 waves (wave 1 – green; waves 2 and 3 – pink). (**b**) Anti-tetanus toxoid IgG levels by visit date (enrolment and delivery; unpaired Welch’s t-test). (**c**) Anti-tetanus toxoid IgG levels at enrolment between first-time pregnancies (**G1**) and second or higher pregnancies (G2+; unpaired Welch’s t-test). (**d**) Anti-tetanus toxoid IgG levels at delivery between first-time pregnancies (**G1**) and second or higher pregnancies (G2+; unpaired t-test). (**e**) Anti-tetanus toxoid IgG levels between pregnancies occurring entirely within the pre-COVID-19 period (solely pre-COVID-19) or pregnancies that occurred exclusively during the Malawi COVID-19 period (solely COVID-19; unpaired t-test). All serological values are shown as log_e_-transformed median fluorescence intensity.

All dates related to the COVID-19 pandemic were obtained from publicly available Malawian Government sources.^5^ Three official waves of COVID-19 hit Malawi through the duration of our trial: COVID-19 wave 1 started in April 2020 and lasted until October 2020. COVID-19 wave 2 started in December 2020 and lasted until the end of April 2021, and COVID-19 wave 3 started in June 2021 and lasted until the end of September 2021.^5^ Government responses were as follows: the first official case of COVID-19 in Malawi was reported on 2 April 2020, and marked the beginning of the first wave of infections (figure 1).^5^ At the request of the Malawi Ministry of Health, REVAMP amended its protocol in May 2020 to reduce visit number and duration.^15^ National lockdown was implemented in January 2021 and COVID-19 vaccination (ChAdOx1-S) began on 11 March 2021.^5^ Vaccination was initially reserved for healthcare workers and made available to the general public in May 2021. In REVAMP, participants were not asked directly about COVID-19 vaccination to avoid retention issues, resulting in incomplete records of COVID-19 vaccinations, though concomitant medication and healthcare visits occurring outside the trial visits were recorded. Malawi experienced three COVID-19 waves during the lifespan of REVAMP.^5^ During the entire trial, there were no self-reported nor trial team-confirmed COVID-19 cases within the cohort and there were no observed or reported positive SARS-CoV-2 tests.

### Serological multiplex bead-array

A multiplex serological assay was used to detect antigen-specific IgG antibodies targeting the SARS-CoV-2 spike protein, spike-derived antigens (S1, S2 and receptor binding domain (RBD)) and nucleoprotein (NP), as previously described.^17^ Additionally, antibodies to seasonal coronavirus antigens (NL63, OC43, HKU1 and 229E) and Influenza virus A (H1N1) were assessed. Antibodies to Tt were also measured as an immunological positive control.^16^ Briefly, individual serum samples were incubated with antigen-coated fluorescent magnetic beads. Antigen-specific antibodies were identified using a secondary anti-human IgG fragment crystallisable antibody conjugated to phycoerythrin and analysed with the MAGPIX (Millipore, Burlington, MA, USA) system. All samples were analysed blinded to participant characteristics.

### Statistical analysis

The sample size was determined by the availability of serum of all randomised women at study enrolment or delivery. Baseline characteristics were summarised and compared before and after COVID-19 emergence in Malawi using either two-sample t or Wilcoxon rank-sum test for continuous data or χ^2^ or Fisher’s exact test for categorical data. Antibody levels were quantified as adjusted mean fluorescent intensity derived from standard curves. Seropositivity for SARS-CoV-2 antibodies was determined as IgG levels exceeding the mean+2 SD from pre-pandemic pregnancies. Longitudinal log_e_-transformed IgG levels were visualised using locally weighted (Locally Weighted Scatterplot Smoothing (LOWESS)) regression with restricted cubic spline curve and 10 smoothing points.^18^ Linear regression models with two inflexion points (start of the COVID-19 pandemic in Malawi on 2 April 2020; beginning of the second COVID-19 wave on 1 January 2021), resulted in three time segments. Models accounted for variables assumed to be prognostic or predictive of the outcomes: maternal age, body mass index, primiparity, sex of the newborn, HIV status, income source and treatment group. Estimates and CIs were back-transformed to reflect the percentage change over 30 days.

Linear regression models were used to quantify associations between pregnancy outcomes of interest (gestational age, birth weight and low birth weight) and serological data at delivery as exposures with adjustment for confounding and prognostic variables—maternal age, primigravidity, body mass index, HIV status and treatment group. Spearman correlations assessed the IgG relationships during the pre-COVID-19 or COVID-19 periods. No adjustment for multiple testing was done.

Small for gestational age was defined as a birth weight below the 10th percentile for gestational age according to The International Fetal and Newborn Growth Consortium for the 21st Century (INTERGROWTH-21) standards.^19^

### Patient and public involvement

This research was performed without participant or public involvement. No patients or members of the public were invited to contribute to the writing or editing of this document.

## Results

### Trial population reflected COVID-19 impacts

The REVAMP trial enrolled 862 participants (Nov 2018–Mar 2021), with the last delivery in the trial occurring in September 2021, thus pregnancies spanned pre-pandemic and pandemic periods (figure 1a). Serum samples from a total of 852/862 (98.8%) women (147 samples from enrolment only, 30 samples from delivery only and 675 samples for both enrolment and delivery) were available and included in this nested study (table 1). Enrolment samples spanned November 2018–March 2021 and delivery samples spanned January 2019–August 2021. Baseline characteristics were similar between women with one sample and those with samples at both time points (online supplemental table 1).

**Table 1:** Baseline characteristics according to the timing of the emergence of COVID-19 in Malawi.

	Before emergence of COVID-19 in Malawi*	After emergence of COVID-19 in Malawi†	Total cohort^¶¶^	Test p value‡
N, %	569 (66.8%)	283 (33.2%)	852 (100%)	
Age (years)	21 (18–26)	19 (17–24)	20 (18–25)	0.0010
BMI§ (kg/m^2^)	22.8 (21.1–24.7)	22.5 (21–24.5)	22.6 (21.1–24.6)	0.20
Primigravid				
No	278 (48.9%)	113 (39.9%)	391 (45.9%)	0.014
Yes	291 (51.1%)	170 (60.1%)	461 (54.1%)	
Religion				
None	0 (0.0%)	2 (0.7%)	2 (0.2%)	0.064
Christian	419 (73.9%)	192 (68.1%)	611 (72.0%)	
Muslim	144 (25.4%)	84 (29.8%)	228 (26.9%)	
Other	4 (0.7%)	4 (1.4%)	8 (0.9%)	
Education				
None	2 (0.4%)	0 (0.0%)	2 (0.2%)	0.0020
Lower primary	98 (17.9%)	74 (27.0%)	172 (21%)	
Upper primary	215 (39.4%)	122 (44.5%)	337 (41.1%)	
Lower secondary	85 (15.6%)	28 (10.2%)	113 (13.8%)	
Upper secondary	132 (24.2%)	45 (16.4%)	177 (21.6%)	
Tertiary	14 (2.6%)	5 (1.8%)	19 (2.3%)	
Marital status				
Single	85 (15%)	47 (16.7%)	132 (15.5%)	0.57
Married	474 (83.6%)	228 (80.9%)	702 (82.7%)	
Widowed	3 (0.5%)	1 (0.4%)	4 (0.5%)	
Divorced/ separated	4 (0.7%)	5 (1.8%)	9 (1.1%)	
Others	1 (0.2%)	1 (0.4%)	2 (0.2%)	
Income source				
None	38 (6.7%)	15 (5.3%)	53 (6.2%)	<0.001
Subsistence farming	74 (13.1%)	73 (25.9%)	147 (17.3%)	
Large scale farming	1 (0.2%)	1 (0.4%)	2 (0.2%)	
Employed	120 (21.2%)	32 (11.3%)	152 (17.9%)	
Casual work for wages	162 (28.6%)	99 (35.1%)	261 (30.7%)	
Business	163 (28.7%)	59 (20.9%)	222 (26.1%)	
Other	9 (1.6%)	3 (1.1%)	12 (1.4%)	
HIV positive				
No	462 (81.8%)	239 (85.4%)	701 (83%)	0.19
Yes	103 (18.2%)	41 (14.6%)	144 (17%)	
Haemoglobin¶ (g/L)	91 (81–98)	87 (79–93)	89 (80–96)	<0.001
Anaemia**				
No	34 (6%)	7 (2.5%)	41 (4.8%)	0.023
Yes	531 (94%)	276 (97.5%)	807 (95.2%)	
Ferritin (µg/L)	25.7 (9.4–64.2)	28.4 (11.6–86.8)	26.8 (10–72.3)	0.95
CRP (mg/L)††	5.3 (2.8–11.1)	5.0 (2.8–9.8)	5.2 (2.8–10.6)	0.85
Inflammation‡‡				
No	269 (47.4%)	137 (51.1%)	406 (48.6%)	0.31
Yes	299 (52.6%)	131 (48.9%)	430 (51.4%)	
Iron deficiency§§				
No	313 (55.1%)	161 (60.1%)	474 (56.7%)	0.18
Yes	255 (44.9%)	107 (39.9%)	362 (43.3%)	

Data are count (%) or median (25% centile, 75% centile)

* Women enrolled in the trial before 2 April 2020

† Women enrolled in the trial on or after 2 April 2020

‡ P values relate to testing for differences between before and after emergence of COVID-19 in Malawi; the analysis included all women with available serological samples at either enrolment or delivery

§ Body mass index

¶ Haemoglobin levels in venous blood, measured by Sysmex

** Anaemia indicates proportion of women with haemoglobin <110 g/L

†† C-reactive protein

‡‡ Inflammation indicates a CRP >5 mg/L

§§ Iron deficient indicates a serum ferritin <15 µg/L, or serum ferritin <30 µg/L if CRP >5 mg/L

¶¶ Total number of women included in the study

BMI, body mass index; CRP, C-reactive protein.

A total of 569 (66.8%) women were enrolled before and 283 (33.2%) women were enrolled after 2 April 2020, the date of the first case of COVID-19 registered in Malawi (table 1), while 358 (42.0%) pregnancies were spent solely under the pre-COVID-19 period (ie, prior to 2 April 2020) and 135 (15.8%) solely under the COVID-19 periods (figure 1a).

We observed differences in demographic characteristics of participants before and after the emergence of SARS-CoV-2 in Malawi (table 1). Timing of participant recruitment revealed maternal age decreased over the course of the trial, from a median of 21 years (IQR (19–27)) in the pre-COVID-19 period to 19 years (IQR (17–23); (p=0.0010)) during the COVID-19 period. Accordingly, we observed an increase in first-time pregnancies towards the end of the trial (51.1% (291/569) pre-COVID-19 vs 60.1% (170/283) COVID; p=0.014). Income source also changed significantly from the pre-COVID-19 to the COVID-19 periods (p<0.001), with more women reporting practising subsistence farming (13.1% (74/567) pre-COVID-19 vs 25.9% (73/282) COVID-19) and fewer women reporting being employed (21.2% pre-COVID-19 vs 11.4% COVID-19).

### Serological outcomes of tetanus toxoid and SARS-CoV-2-specific antibodies

We measured antibodies to Tt as a validation tool for our serological assays. As was expected from Malawi’s vaccination policy,^16^ Tt-specific IgG levels were higher at delivery versus enrolment (figure 1b), and in women with two or more pregnancies compared with women in their first pregnancy—both at enrolment and, though to a lesser degree, at delivery (figure 1c–d). The overall levels of anti-Tt antibodies at delivery were unaffected in those pregnancies carried solely within the COVID-19 period versus those solely in the pre-COVID-19 period (figure 1e).

**Figure 2:**
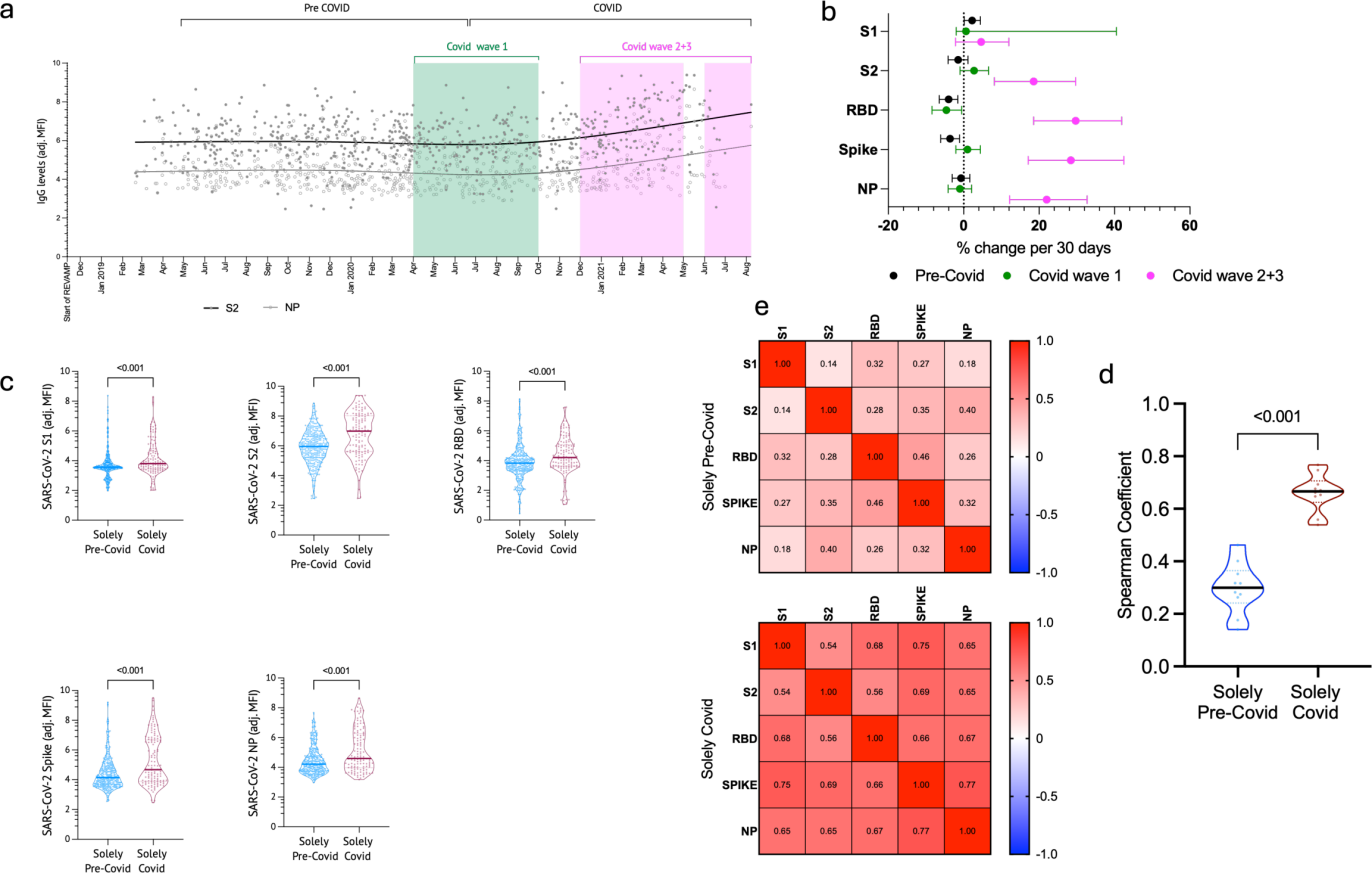
Serological patterns of anti-SARS-CoV-2 antibodies during pregnancy. (**a**) locally estimated scatterplot smoothing (LOWESS) smoothing curves illustrating the relationship between time and anti-SARS-CoV-2 antibodies is shown for SARS-CoV-2 S2 and SARS-CoV-2 nucleoprotein (NP) antigens. The analysis was conducted using GraphPad Prism V.10, Software,San Diego, CA, USA. This method fits a non-parametric regression and makes no assumptions about the functional form of the relationship. The graph includes the original data points as scatter markers to allow assessment of the density and distribution of observations across the range of values. (**b**) Mean percent change in IgG levels every 30 days across the three time periods assessed after fitting a linear regression model. (**c**) Comparison of mean antibody levels to SARS-CoV-2 antigens between pregnancies occurring outside (entirely pre-COVID-19) or inside (entirely COVID-19) the COVID-19 period (unpaired t-test). (**d**) Correlation coefficient matrix between antibodies measured against SARS-CoV-2 antigens in samples from pregnancies outside (solely pre-COVID-19 – top) or inside (solely COVID-19 – bottom) the COVID-19 period. (**e**) Comparison of mean correlation coefficient between antibodies against SARS-CoV-2 antigens between pregnancies occurring outside (solely pre-COVID-19) or inside (solely COVID-19) the COVID-19 period (unpaired t-test).

Antibodies to all five SARS-CoV-2 antigens at delivery increased from the beginning of 2021, coinciding with the emergence of COVID waves 2 and 3 in Malawi (figure 2a and online supplemental figure 1). It should be noted that IgG levels to SARS-CoV-2 antigens were detected even prior to the pandemic, indicating the occurrence of cross-reactive immunity in this population. In general, there was a subtle positive trend in antibody levels over time, with values remaining relatively stable to the end of COVID-19 wave 1, followed by an increase through COVID-19 waves 2 and 3. Linear modelling of this relationship revealed a significant increase in antibodies to SARS-CoV-2 antigens at delivery during COVID-19 waves 2 and 3 (figure 2b, online supplemental table 2). This increase was also noticeable when comparing mean antibody levels in those pregnancies carried solely within the COVID-19 period versus those occurring in the pre-COVID-19 period (figure 2c). In addition, the correlations between IgG levels to all SARS-CoV-2 antigens also significantly increased from those pregnancies entirely in the pre-COVID-19 period to those entirely in the COVID-19 period (figure 2e–d).

A total of 102 delivery samples (17.3%) were seropositive for at least one of the SARS-CoV-2 antigens (ranging from 4.3% for RBD to 9.1% against spike). Half of the 102 seropositive samples (49%) were seropositive for 1 antigen and only 5 samples (4.1%) were seropositive for all 5 SARS-CoV-2 antigens. The overall seroprevalence calculated for those pregnancies entirely outside of the COVID-19 period in Malawi was 14.7% (48/327), revealing a significant number of cross-reactive responses to other antigens. In pregnancies occurring entirely within the COVID-19 period, the overall seroprevalence was 39.3% (48/122).

### Serological outcomes – seasonal coronaviruses and Influenza A

**Figure 3:**
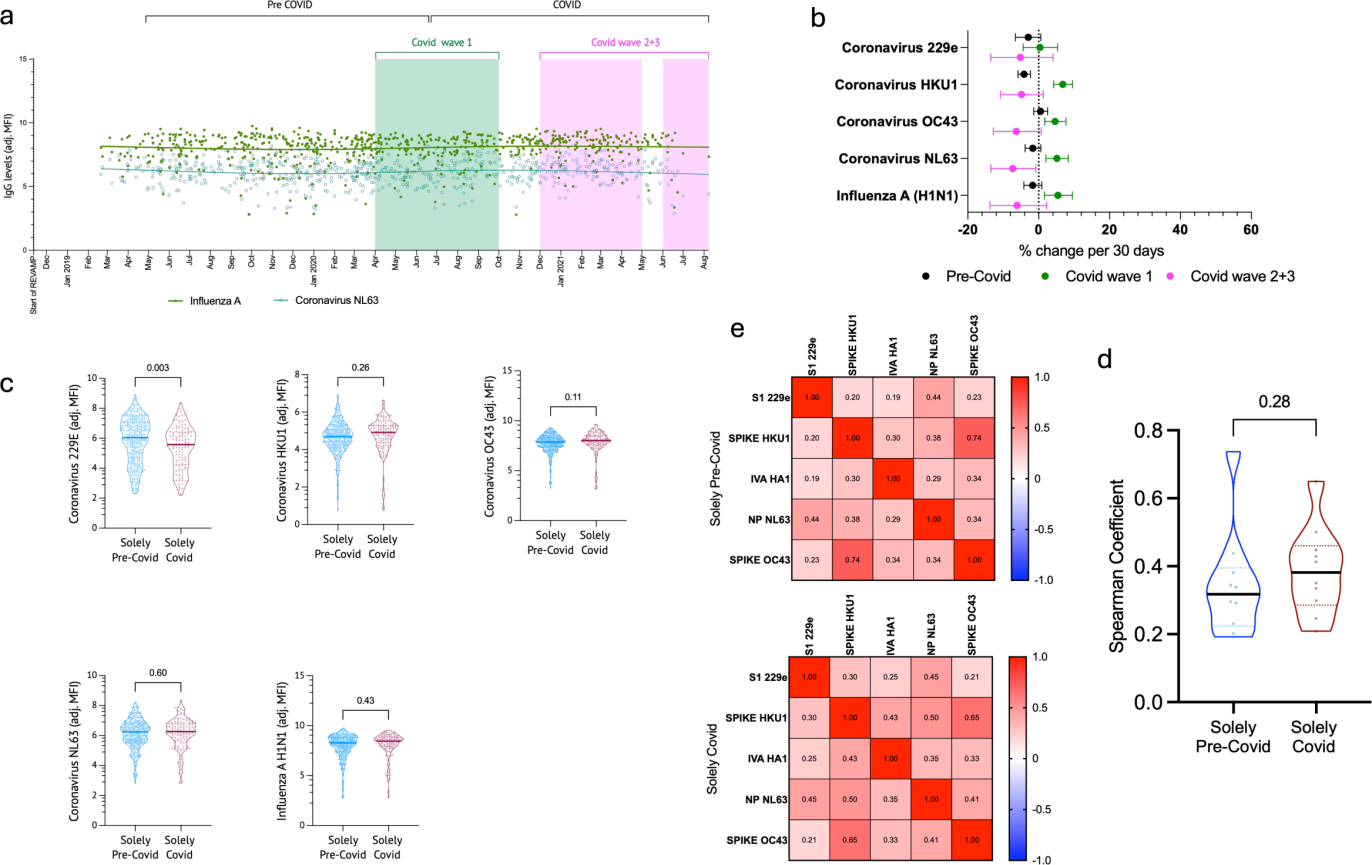
Serological patterns of anti-common coronavirus and Influenza A (H1N1) antibodies during pregnancy. (**a**) locally estimated scatterplot smoothing (LOWESS) smoothing curves illustrating the relationship between time and antibodies against Influenza A (green) and coronavirus NL63 (teal) antigens. The analysis was conducted using GraphPad Prism V.10, GraphPad Software, San Diego, CA, USA. This method fits a non-parametric regression and makes no assumptions about the functional form of the relationship. The graph includes the original data points as scatter markers to allow assessment of the density and distribution of observations across the range of values. (**b**) Mean percent change in IgG levels every 30 days across the three time periods assessed after fitting a linear regression model. (**c**) Comparison of mean antibody levels to seasonal coronavirus and Influenza A antigens between pregnancies occurring outside (entirely pre-COVID-19) or inside (entirely COVID-19) the COVID-19 period (unpaired t-test). (**d**) Correlation coefficient matrix between antibodies measured against seasonal coronavirus antigens in samples from pregnancies outside (solely pre-COVID-19 – top) or inside (solely COVID-19 – bottom) the COVID-19 period. (**e**) Comparison of mean correlation coefficient between antibodies against seasonal coronavirus antigens between pregnancies occurring outside (solely pre-COVID-19) or inside (solely COVID-19) the COVID-19 period (unpaired t-test).

At delivery, antibodies to seasonal viruses presented a distinct pattern across the timeline of the trial (figure 3a–b and online supplemental figure 1). LOWESS visualisation showed little variation across the entirety of the trial (figure 3a). Linear modelling over the three segments of the trial revealed a general stability over the pre-COVID-19 period—maximum variation was for IgG towards coronavirus HKU1 (mean % change over 30 days: −4.1% (95% CI −5.8 to −2.3); online supplemental table 2). This was followed by a small increase in IgG levels during the second period, and an overall decrease during the last period (figure 3b, online supplemental table 2). However, the levels of antibodies to these common human coronaviruses, including types NL63, OC43 and HKU1, and to Influenza A (H1N1) remained relatively unchanged when analysing those pregnancies occurring outside or inside the COVID-19 period, except for antibodies to coronavirus 229E that declined significantly at delivery between solely pre-COVID-19 and solely COVID-19 pregnancies (figure 3c). There was no significant change to the correlations between antigens to the common coronaviruses and Influenza A between COVID-19 periods (figure 3c–d).

### Pregnancy outcomes of the trial according to COVID-19 period and association with SARS-CoV-2 antibodies

Throughout the trial, and after accounting for confounding variables, gestation duration increased significantly between pregnancies that delivered during the COVID-19 period compared with pre-COVID-19 (adjusted mean difference 0.5 weeks (95% CI 0.2 to 0.8); table 2). This difference was still observed when looking at women with pregnancies entirely in the COVID-19 period versus entirely in the pre-COVID-19 period (mean difference 0.6 weeks (95% CI 0.2 to 0.9)). Accordingly, babies had higher birth weights when delivered during the COVID-19 period compared with those delivered during the pre-COVID-19 period (mean difference 69.4 g (95% CI −2.4 to 141.1)), although this increased birth weight was only significant when comparing those pregnancies occurring solely within the COVID-19 period versus occurring solely in pre-COVID-19 (mean difference 169.3 g (95% CI 65.9 to 272.6)). Low birth weight decreased during the COVID-19 period (OR COVID-19 vs pre-COVID-19: 0.57 (95% CI 0.37 to 0.89)), and in those pregnancies solely occurring during COVID-19 versus in pre-COVID-19 (OR solely COVID-19 vs pre-COVID-19: 0.40 (95% CI 0.20 to 0.77); table 2).

**Table 2:** Comparison of pregnancy and birth outcomes among women enrolled in the REVAMP study stratified by whether delivery or the entire gestation occurred before or after first known case of COVID-19 in Malawi

Delivery inside versus outside COVID-19	Pre-COVID-19 before 2 April 2020	COVID-19 after 2 April 2020	OR (95% CI) or mean difference (95% CI)	P value
Small for gestational age n/N (%)	146/477 (30.6%)	92/266 (34.6%)	1.10 (0.79 to 1.53)	0.57
Low birth weight (<2500 g) n/N (%)	89/484 (18.4%)	32/265 (12.1%)	0.57 (0.37 to 0.89)	0.013
Premature birth (<37 weeks) n/N (%)	43/489 (8.8%)	17/270 (6.3%)	0.70 (0.39 to 1.26)	0.23
Fetal loss n/N (%)	11/522 (2.1%)	5/275 (1.8%)	0.95 (0.32 to 2.82)	0.93
Gestation duration (weeks) mean (SD)*	39.3 (2.1)	39.8 (1.9)	0.49 (0.20 to 0.78)	0.0010
Birth weight (g) mean (SD)†	2889.9 (501.4)	2930 (477.8)	69.36 (−2.39 to 141.12)	0.06
Entire gestation period inside versus outside COVID-19				
Small for gestational age n/N (%)	94/335 (28.1%)	40/130 (30.8 %)	0.97 (0.62 to 1.53)	0.90
Low birth weight (<2500 g) n/N (%)	64/340 (18.8%)	12/127 (9.4%)	0.40 (0.20 to 0.77)	0.0070
Premature birth (<37 weeks) n/N (%)	35/343 (10.2%)	8/130 (6.2%)	0.59 (0.26 to 1.33)	0.20
Fetal loss n/N (%)	5/350 (1.4%)	1/132 (0.8%)	0.53 (0.05 to 5.19)	0.59
Gestation duration (weeks) mean (SD)‡	39.2 (2.2)	39.8 (1.6)	0.6 (0.2 to 0.9)	0.0030
Birth weight (g) mean (SD)§	2883.2 (516.3)	3001.5 (487.4)	169.3 (65.9 to 272.6)	0.0010

Note: small for gestational age defined as a birth weight below the 10th percentile for gestational age according to INTERGROWTH-21 standards^19^; low birth weight is defined as a birth weight <2500 g; premature birth is a birth occurring at <37 weeks’ gestation; fetal loss represents a composite of pregnancy loss and stillbirth

All models controlled for treatment group, maternal age, body mass index, primigravid status and HIV status; analyses were run on all samples with non-missing data

* n=489 pre-COVID-19 and 270 COVID-19

† n=484 pre-COVID-19 and 265 COVID-19

‡ n=343 pre-COVID-19 and 130 COVID-19

§ n=340 pre-COVID-19 and 127 COVID-19

In women whose pregnancies occurred within the COVID-19 period, we found no evidence of a statistically significant association between seropositivity to SARS-CoV-2 antigens at delivery and pregnancy outcomes. However, the estimates around the associations had very large 95% CI, which is compatible with large effects in either direction (online supplemental table 3).

## Discussion

The REVAMP trial conducted continuous sampling throughout pregnancy, and up to delivery, from women before the emergence of the COVID-19 pandemic and up to the height of the 2021 COVID-19 waves in Malawi. We observed significant demographic shifts throughout the duration of the trial where women enrolled during the pandemic were significantly younger than those enrolled in the pre-COVID-19 period. This change was accompanied by significant changes to the parity, education and income source measures of the trial cohort—reflecting the younger population. This observed shift in population age may reflect broader pandemic-related socio-economic changes, as Malawi saw overall health service use drop 10% and antenatal visits 3% from April 2020 to December 2021.^20^ It is reasonable to assume that Malawian government enforced lockdowns, school closures and other containment measures^5^ likely limited participation and healthcare access for women with young children as they may have had responsibilities that prevented them from either participating in the study or engaging with the health services. These findings underscore the need to consider population characteristics in long-term studies.

During the REVAMP trial, women were assessed by a clinician at each scheduled visit (including enrolment, 4 weeks post-enrolment, 36 weeks’ gestation and delivery). All assessments began with asking the women whether they had any symptoms or illness that day, followed by a physical examination. In addition, women were also asked whether they had experienced any symptoms or had any medical treatment since their previous visit. Among the reported symptoms or illnesses, we had no suspected COVID-19 case or registered the occurrence of a positive SARS-CoV-2 test. Importantly, at the peak of the pandemic, our clinical staff heightened the vigilance in screening. In this study, SARS-CoV-2-specific antibody levels significantly increased over the study period in pregnant women lacking any evidence of clinical symptoms consistent with COVID-19 or laboratory-confirmed positive SARS-CoV-2 tests. This increase was particularly noted during the second and third COVID-19 waves in Malawi. Our serological findings contribute to understanding immune responses to SARS-CoV-2 in African pregnant women. A meta-analysis of the seroprevalence of anti-SARS-CoV-2 antibodies across Africa (excluding Malawi) revealed seroprevalences ranging from 0% to 63%,^21^ while studies in Malawi estimated prevalences at between 10% and 85% depending on the sampling strategy and the population.^6 22 23^ In REVAMP, we measured a peak seroprevalence of 39.3%. Although seropositivity to SARS-CoV-2 does not immediately follow from increases in seroprevalence to single antigens, the increase of antibody levels to all five SARS-CoV-2 antigens during the COVID-19 period, coupled with stronger inter-antigenic correlations, suggests true community transmission occurred in the trial population during this period. This timing agrees with SARS-CoV-2 seroprevalence data obtained from blood donations surveyed from four distinct areas of Malawi^6^ and from pregnant women at first antenatal clinic in a country-wide study, which started just as REVAMP was recruiting its last participants.^23^

Importantly, the rise and timing of IgG levels aligned with seroprevalence trends reported across Africa in several different populations,^24^ highlighting the reliability of pregnant women as a sentinel population (online supplemental figure 2). The overall rise in antibody levels to all SARS-CoV-2 antigens evaluated suggests that this was due to community transmission of the virus, rather than through active vaccination campaigns, which only started in March 2021 and experienced low uptake until the end of 2021.^5 24^ Additionally, given that ChAdOx1-S vaccine used the SARS-CoV-2 spike protein only, the increase in antibodies to non-S protein antigens within this study population would only be expected to be generated following SARS-CoV-2 infection.^25^ Notably, in the year before the emergence of SARS-CoV-2, the degree of background reactivity we detected—not observed in other cohorts^17^—raises questions about potential immunological cross-reactivity between SARS-CoV-2 antigens and other antigens in this population. The clinical significance of this observation remains to be determined.

IgG levels measured against common seasonal coronaviruses as well as Influenza A revealed distinct patterns to those observed against all SARS-CoV-2 antigens, and remained consistent across the trial period. The stability of Tt antibody levels across all periods served as an important internal control, confirming the specificity of SARS-CoV-2 antibody changes. As Tt vaccinations were government-provided, this also indicates that antenatal care attendance and services remained relatively robust despite COVID-19-related disruptions in this region of Malawi.^20^

Despite the serological evidence of increasing SARS-CoV-2 exposure in the population, pregnancies during the COVID-19 period of the trial had longer gestations and higher birth weights. As with many respiratory infections,^26^ SARS-CoV-2 infection has been reported to negatively affect birth outcomes.^27^ However, observations of better outcomes for pregnancy during the pandemic have been made in other cohorts as well.^28^ These results suggest that SARS-CoV-2 exposure was either too low to affect outcomes, or its negative effects were offset by reductions in other infections, or improvements reflected maternal changes with reduced mobility and physical demands that may result in conserved maternal energy and greater partner presence providing added support during pregnancy.

These findings, while strengthened by standardised trial follow-up, may not apply to the broader Malawian population. We did not collect specific information regarding COVID-19 vaccination status of our participants, and it is possible that some women in the cohort could have been vaccinated against SARS-CoV-2 from May 2021 onward, when vaccines were made available to the wider public. The timing of the study makes it challenging to disentangle the effects of SARS-CoV-2 infection from the effects of pandemic-related social and healthcare changes. Although our models accounted for shifting demographics, such as age and primiparity, the demographic changes could have introduced unmeasured confounding factors not accounted for in our statistical adjustments. Additionally, it is important to note that with regular antenatal care and supplementation, trial participants may have been relatively protected from pandemic impacts compared with the general population.^20^

In conclusion, we demonstrate that in a cohort of pregnant women in Malawi enrolled in the REVAMP clinical trial, who did not present with or were reported throughout pregnancy to have symptoms of COVID-19 or SARS-CoV-2 infection, population-level IgG to SARS-CoV-2 antigens (both vaccine-related and vaccine-independent) rose in alignment with well-defined COVID-19 periods. Additionally, the increase in inter-antigenic correlation between the antibody levels to SARS-CoV-2 antigens during the COVID-19 period also sustains the interpretation that there was true population transmission during the COVID-19 pandemic. This represents additional evidence to the body of research that proposes pregnancy as a sensitive sentinel period for the population surveillance of infectious diseases.^11 12 29 30^

## Supplementary material

10.1136/bmjopen-2025-113123 online supplemental file 1

## Data Availability

Data are available upon reasonable request.
