# Supplementary material for: Longitudinal serological detection of exposure to SARS-CoV-2 in a cohort of pregnant women in Malawi: a secondary analysis from a randomised controlled trial
Source: BMJ Open. 2026 Jun 29;16(6):e113123. doi: 10.1136/bmjopen-2025-113123 (PMC13331037; doi:10.1136/bmjopen-2025-113123)
Supplement: Supplementary file 2 [file bmjopen-2025-113123.reviewer_comments.pdf]

PEER REVIEW HISTORY

BMJ Open publishes all reviews undertaken for accepted manuscripts. Reviewers are asked to complete a checklist review form and are provided with free text boxes to elaborate on their assessment. These free text comments are reproduced below.

ARTICLE DETAILS

Title (Provisional)

Longitudinal serological detection of exposure to SARS-CoV-2 in a cohort of pregnant women in Malawi: A secondary analysis from a randomized controlled trial.

Authors

Randall, Louise; Kiernan-Walker, Nicholas; Moya, Ernest; Mzembe, Glory; McLean, Alistair R.D.; Harding, Rebecca; Mazhari, Ramin; Mhango, Gomezgani; Fielding, Katherine L; Mueller, Ivo; Mwangi, Martin N.; Braat, Sabine; Phiri, Kamija; Pasricha, Sant-Rayn; Eriksson, Emily M; Ataíde, Ricardo

VERSION 1 - REVIEW

**Reviewer**	**1**
**Name**	**DeSilva, Malini B.**
**Affiliation**	**HealthPartners Institute for Medical Education, Research**
**Date**	**07-Nov-2025**
**COI**	**None**

This manuscript attempted to determine whether monitoring of IgG to SARS-CoV-2 antigens could reveal undetected SARS-CoV-2 exposure in Malawian pregnant women. While the methods are able to show antibody levels which may correlate with SARS-CoV-2 exposure, these levels may also be related to vaccination or as noted by the authors cross-reactivity with a different pathogen. The authors conclusions go too far in suggesting the spread of COVID-19 in this population based solely on antibody levels while not reporting if they collected symptoms from patients or vaccination information. Being clear in the exact time periods studied and how these time periods corresponded to various time points is needed. Below are some specific comments:

- Line 96 – "…among other" it would be helpful to know hat other criteria were required, may list in supplement or refer to other work as well

- Line 97 – Does "prevalence at 36 weeks" refer to 36 weeks gestation or 36 weeks following treatment?

- Lines 97 – 98, Please provide more information about exact timing in this sentence, “Venous blood was collected at enrolment (13–26 weeks), during pregnancy, and at delivery” was this 13 – 26 weeks gestation?, was there only one other time during pregnancy or at 36 weeks gestation only?

- Line 128 – I assume Tt is tetanus toxoid? It would be good to put this in parentheses the first time it is used in the manuscript

- Table 2 – It would be helpful to change the title of the table, something along the lines of “Comparison of pregnancy and birth outcomes among women enrolled in the REVAMP study stratified by whether delivery occurred before or after first known case of COVID-19 in Malawi” To that end, it would also be helpful to add the time periods to the footnote at least. Finally, I do not see the footnote annotations in the table, please add these so this is more clear.

- Line 267 - The discussion notes that antibody levels increased, “in pregnant women lacking clinical symptoms and laboratory-confirmed positive tests” however this data is not presented. Were participants asked about COVID-19 symptoms? If not, this needs to be clearly stated and any allusions to asymptomatic pregnant women needs to be removed as this is not certain.

- The biggest change in antibody levels occurred from COVID wave 2 to COVID wave 3. The 2nd COVID wave started 1.1.21 and vaccinations started 3.11.21 in Malawi, thus, vaccines in addition to natural infection may have been part of this increase in antibody levels. The conclusions should acknowledge the limitations of the analysis and what is not known including how many of the pregnancy women received the vaccine.

- The exact dates of the COVID-19 waves are not clear, please explicitly state these

---

**Reviewer**	**2**
**Name**	**Mullins, Edward**
**Affiliation**	**Imperial College London, Reproductive Medicine**
**Date**	**11-Feb-2026**
**COI**	**None**

Thank you for this paper making secondary use of a pregnancy cohort study into iron deficiency to determine seroprevalence of SARS-CoV-2.

I couldn't determine if the seroprevalence data presented for the antibodies to SARS-CoV-2 comprised assays on the early pregnancy samples, delivery samples or both.

Why was an assay used which had a false positive rate of 14%? (ie positive on samples from prior to the pandemic). Was consideration given to confirmatory testing with a different assay given this high FP rate e.g. neutralising antibodies or one of the commercial platforms?

Were the early samples compared to estimate how many women with early and delivery samples were exposed to SARS-CoV-2 during pregnancy?

Figure 1 and 2 came through on the proof as coloured shapes with no axes or labels, so I could not interpret them.

Overall, as a concept this is a useful example of serendipitous repurposing of a trial during a pandemic. I agree maternal populations are likely to be useful during a pandemic as sentinel populations as they have interactions with, depending on the societal structure of the area studied, children, adults and the elderly.

My group at Imperial College London used routine, stored maternal sera in London to study the onset of community transmission of SARS CoV 2, which alongside more deeply phenotyped research cohorts from multiple countries would allow a network of sentinel populations to be activated at short notice when reliable assays become available. We are actively looking for collaborators.

VERSION 1 - AUTHOR RESPONSE

Reviewer #1

Dr. Malini B. DeSilva, HealthPartners Institute for Medical Education

Thank you to Dr DeSilva for taking the time to read the manuscript and allowing us the opportunity to improve it.

This manuscript attempted to determine whether monitoring of IgG to SARS-CoV-2 antigens could reveal undetected SARS-CoV-2 exposure in Malawian pregnant women. While the methods are able to show antibody levels which may correlate with SARS-CoV-2 exposure, these levels may also be related to vaccination or as noted by the authors cross-reactivity with a different pathogen. The authors conclusions go too far in suggesting the spread of COVID-19 in this population based solely on antibody levels while not reporting if they collected symptoms from patients or vaccination information. Being clear in the exact time periods studied and how these time periods corresponded to various time points is needed.

Response:

Thank you. The reviewer is correct in pointing out that we are unable to verify if the increase in antibodies detected against SARS-CoV-2 seen from the beginning of 2021 is simply the result of cross-reactivity against an unmeasured pathogen, however we would like to argue that vaccination to SARS-CoV-2 is an unlikely source. As mentioned, the vaccines arrived in Malawi in March of 2021, however, vaccination was initially limited to healthcare workers until May 2021, when it was made available to the general public. As can be observed in our data, the increase in antibody levels in our cohort was in effect much earlier than that. In addition, the vaccine available in Malawi was the Oxford-AstraZeneca

ChAdOx1-S, which elicits responses to epitopes on the Spike protein of SARS-CoV-2, whilst we observed an increase in antigens including the nucleoprotein (NP) that would not have occurred outside of natural infection under the circumstances in Malawi at the time of the study. This was the reasoning leading us to conclude that natural exposure to SARS-CoV-2 was responsible for the increase in IgG levels observed.

We have clarified the time period for collection of the available samples for this study. In the results section we have included the following:

"**Enrolment samples spanned November 2018-March 2021 and delivery samples spanned from January 2019-August 2021**."

Q: Line 96 – "…among other" it would be helpful to know that other criteria were required, may list in supplement or refer to other work as well

Response:

Thank you for noticing the omission of the reference. We have now added the correct reference to the end of that sentence to direct the reader to the study protocol of the REVAMP trial, where detailed inclusion and exclusion criteria are listed.

Q: Line 97 – Does "prevalence at 36 weeks" refer to 36 weeks gestation or 36 weeks following treatment?

Response:

We have clarified that sentence to highlight it was 36 weeks' gestation. It now reads:

"**The primary endpoint of the REVAMP trial was anaemia prevalence at 36 weeks' gestation**."

Q: Lines 97 – 98, Please provide more information about exact timing in this sentence, "Venous blood was collected at enrolment (13–26 weeks), during pregnancy, and at delivery" was this 13 – 26 weeks gestation?, was there only one other time during pregnancy or at 36 weeks gestation only?

Response:

Thank you. We have decided to clarify the timepoints of collection of samples that were used in this study. Although the REVAMP trial collected samples across pregnancy, the samples used in the current analyses are from enrolment and delivery only. That sentence in the Study design section now reads:

"**Venous blood samples used in this study were collected at enrolment (13–26 weeks' gestation), and at delivery**."

Q: Line 128 – I assume Tt is tetanus toxoid? It would be good to put this in parentheses the first time it is used in the manuscript.

Response:

Thank you for pointing that out. We have added (Tt) to the first instance of Tetanus toxoid in the manuscript.

Q: Table 2 – It would be helpful to change the title of the table, something along the lines of "Comparison of pregnancy and birth outcomes among women enrolled in the REVAMP study stratified by whether delivery occurred before or after first known case of COVID-19 in Malawi" To that end, it would also be helpful to add the time periods to the footnote at least. Finally, I do not see the footnote annotations in the table, please add these so this is more clear.

Response:

Thank you for that suggestion. It does make the table clearer and more informative. We have changed the name of the table to: "**Comparison of pregnancy and birth outcomes among women enrolled in the REVAMP study stratified by whether delivery or the entire gestation occurred before or after first known case of COVID-19 in Malawi**". Additionally, we have added the time periods to the top of the table to make it explicit.

Regarding the footnote table annotations, does the reviewer mean the highlighted below? Apologies if we misunderstood.

Gestation duration (weeks) Mean (SD)#	39.3 (2.1)	39.8 (1.9)	0.49 [0.20-0.78]	0.0010
Birth weight (grams) Mean (SD)$	2889.9 (501.4)	2930 (477.8)	69.36 [-2.39-141.12]	0.06

Q: Line 267 - The discussion notes that antibody levels increased, "in pregnant women lacking clinical symptoms and laboratory-confirmed positive tests" however this data is not presented. Were participants asked about COVID-19 symptoms? If not, this needs to be clearly stated and any allusions to asymptomatic pregnant women needs to be removed as this is not certain.

Response:

Thank you for the opportunity to clarify this important point. On each scheduled visit, the women were assessed by a clinician. The assessment began with asking them whether they had any symptoms or illness that day, followed by a physical examination. In addition to symptom screening on the specific day, the women were also asked whether they had experienced any symptoms or had any medical treatment since their previous visit. All women who reported any symptoms received treatment accordingly, and the information was captured on the adverse events form. Among the reported symptoms or illnesses, we had no suspected COVID-19 case and did not refer any of the participants for testing. Importantly, at the peak of the pandemic, there was heightened vigilance in screening.

However, and as acknowledged in the Limitations section, the REVAMP trial did not capture specific COVID-19 data, nor specifically asked about COVID-19 to the participants.

We have attempted to make this clearer throughout the discussion.

"**During the REVAMP trial, women were assessed by a clinician at each scheduled visit (including enrolment, 4 weeks post enrolment, 36 weeks' gestation and delivery). All assessments began with asking the women whether they had any symptoms or illness that day, followed by a physical examination. In addition, women were also asked whether they had experienced any symptoms or had any medical treatment since their previous visit. Among the reported symptoms or illnesses, we had no suspected COVID-19 case or registered the occurrence of a positive SARS-CoV-2 test. Importantly, at the peak of the pandemic, our clinical staff heightened the vigilance in screening.**"

"In conclusion, we demonstrate that in a cohort of pregnant women in Malawi enrolled in the REVAMP clinical trial, who did not present with or were reported throughout pregnancy to have symptoms of COVID-19 or SARS-CoV-2 infection, population-level IgG to SARS-CoV-2 antigens (both vaccine-related and vaccine-independent) rose in alignment with well-defined COVID-19 periods."

Q: The biggest change in antibody levels occurred from COVID wave 2 to COVID wave 3. The 2nd COVID wave started 1.1.21 and vaccinations started 3.11.21 in Malawi, thus, vaccines in addition to natural infection may have been part of this increase in antibody levels. The conclusions should acknowledge the limitations of the analysis and what is not known including how many of the pregnancy women received the vaccine.

Response:

We understand the reviewer's concerns regarding the potential influence of vaccination on the increase of IgG levels to SARS-CoV-2 seen in our cohort. As we highlighted above, though, we have data to support the unlikeliness of that being the case. Just to summarise once more:

1- Vaccines arrived in Malawi in March of 2021, however, vaccination was initially limited to healthcare workers until May 2021, when it was made available to the general public. As can be observed in our data, the increase in antibody levels in our cohort was in effect from January of 2021, a full 4 months before the vaccine became available to the wider public.
2- The vaccine available in Malawi was the Oxford-AstraZeneca ChAdOx1-S, which elicits responses to epitopes on the Spike protein of SARS-CoV-2. We observed an increase in IgG response to antigens (such as the NP) that would not have occurred outside of natural infection.

This is the reasoning that leads us to conclude that natural exposure to SARS-CoV-2, rather than vaccination campaigns, is the best explanation for the increase in IgG levels observed in this cohort. We do not discount that levels of IgG from May to September may include women who were vaccinated.

We have added the following to the discussion:

"We did not collect specific information regarding COVID-19 vaccination status of our participants, **and it is possible that some women in the cohort could have been vaccinated against SARS-CoV-2 from May 2021 onward, when vaccines were made available to the wider public**."

Q: The exact dates of the COVID-19 waves are not clear, please explicitly state these.

Response:

We have added the following text (based on the official information from the Malawian Government, and reflected in our figures and tables), to the COVID-19 Setting section of the manuscript:

"Three official waves of COVID-19 hit Malawi through the duration of our trial: COVID-19 wave-1 started in April 2020 and lasted until October 2020. COVID-19 wave-2 stated in December 2020 and lasted until the end of April 2021 and COVID-19 wave-3 started in June 2021 and lasted until the end of September 2021."

Reviewer #2

Dr. Edward Mullins, Imperial College London

Thank you to Dr Mullins for the time taken to read through our manuscript and provide us with comments.

Comments to the Author:

Thank you for this paper making secondary use of a pregnancy cohort study into iron deficiency to determine seroprevalence of SARS-CoV-2.

Q: I couldn't determine if the seroprevalence data presented for the antibodies to SARS-CoV-2 comprised assays on the early pregnancy samples, delivery samples or both.

Response:

Thank you for allowing us to clarify this issue. We measured IgG levels in this cohort at enrolment (second trimester) and at delivery. All the analysis was conducted with the Delivery antibody samples (as a proxy to the full history of exposure during pregnancy). Enrolment samples were used to validate the use of serology in this cohort (as antibodies to Tt should have been significantly lower in the cohort at enrolment versus at delivery) and to assess the possibility of women becoming SARS-CoV-2 positive during their time on trial or having arrived on trial already positive. We have added several instances of "at delivery" throughout the text to clarify this.

Q: Why was an assay used which had a false positive rate of 14%? (ie positive on samples from prior to the pandemic). Was consideration given to confirmatory testing with a different assay given this high FP rate e.g. neutralising antibodies or one of the commercial platforms?

Response:

Thank you to the reviewer for this observation and an opportunity to clarify. The serological assay used for this study was chosen as it offered a high throughput assay in which multiple antigen-specific antibodies could be measured from a small volume of sample.[1] This assay had also has been extensively tested on populations from Australia where it demonstrated an extremely low false-positive rate. Furthermore, this assay was validated against the Wantai SARS-CoV-2 Ab test. The Wantai SARS-CoV-2 Ab test is an accredited assay that measures antibodies to SARS-CoV-2 at high specificity (97.5%) and sensitivity (96.7%) and was commonly used for serosurveillance during the pandemic. As part of our validation when developing the current assay, we correlated our Luminex assay MFI read-outs to the OD450nm readouts of the Wantai ELISA kit showing a high positive Spearman correlation ($r=0.9$; $p<0.0001$) of the two assays. The finding of 14% seroprevalence prior to the emergence of SARS-CoV-2 is a clear indication to us of the necessity of taking the baseline study population's antigen exposure and immune landscape into consideration when evaluating serological data.

[1] Mazhari R, Ruybal-Pesántez S, Angrisano F, *et al.* SARS-CoV-2 Multi-Antigen Serology Assay. *Methods Protoc* 2021; **4**. DOI:10.3390/mps4040072.

Q: Were the early samples compared to estimate how many women with early and delivery samples were exposed to SARS-CoV-2 during pregnancy?

Response:

Great question. Yes, we did compare the levels of antibodies at enrolment and at delivery and found that all positive women at delivery had become seropositive during pregnancy, rather than sustained a seropositive result throughout pregnancy.

Q: Figure 1 and 2 came through on the proof as coloured shapes with no axes or labels, so I could not interpret them.

Response:

We apologise! We have included those figures in this document in the hope that any formatting errors will not occur again and the reviewer can clearly evaluate them. Please see below.

Q: Overall, as a concept this is a useful example of serendipitous repurposing of a trial during a pandemic. I agree maternal populations are likely to be useful during a pandemic as sentinel populations as they have interactions with, depending on the societal structure of the area studied, children, adults and the elderly.

Response:

Thank you to the reviewer for recognising the usefulness of this population as a potential sentinel. This is an important observation that deserves to be fully investigated.

Q: My group at Imperial College London used routine, stored maternal sera in London to study the onset of community transmission of SARS CoV 2, which alongside more deeply phenotyped research cohorts from multiple countries would allow a network of sentinel populations to be activated at short notice when reliable assays become available. We are actively looking for collaborators.

Response:

Thank you to the reviewer for the willingness to extend his collaborative network. We would be interested to contact the reviewer and discuss this possibility once the review process has followed its proper course.

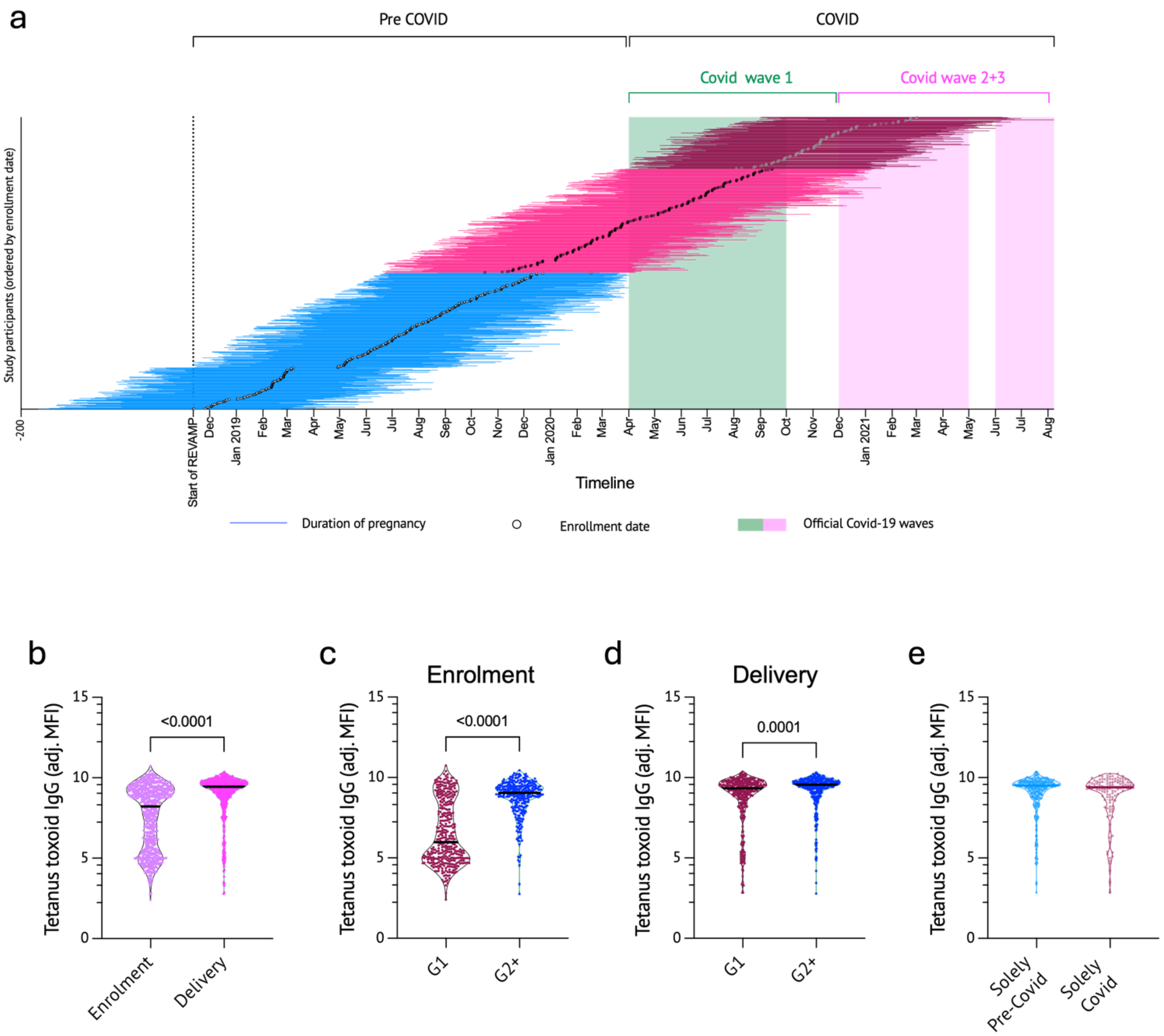
a
Pre COVID
COVID
Covid wave 1
Covid wave 2+3
Study participants (ordered by enrollment date)
-200
Start of REVAMP
Dec
Jan 2019
Feb
Mar
Apr
May
Jun
Jul
Aug
Sep
Oct
Nov
Dec
Jan 2020
Feb
Mar
Apr
May
Jun
Jul
Aug
Sep
Oct
Nov
Dec
Jan 2021
Feb
Mar
Apr
May
Jun
Jul
Aug
Timeline
Duration of pregnancy
Enrollment date
Official Covid-19 waves
b
Tetanus toxoid IgG (adj. MFI)
15
10
5
0
<0.0001
Enrolment
Delivery
c
Enrolment
Tetanus toxoid IgG (adj. MFI)
<0.0001
G1
G2+
d
Delivery
Tetanus toxoid IgG (adj. MFI)
0.0001
G1
G2+
e
Tetanus toxoid IgG (adj. MFI)
Solely Pre-Covid
Solely Covid

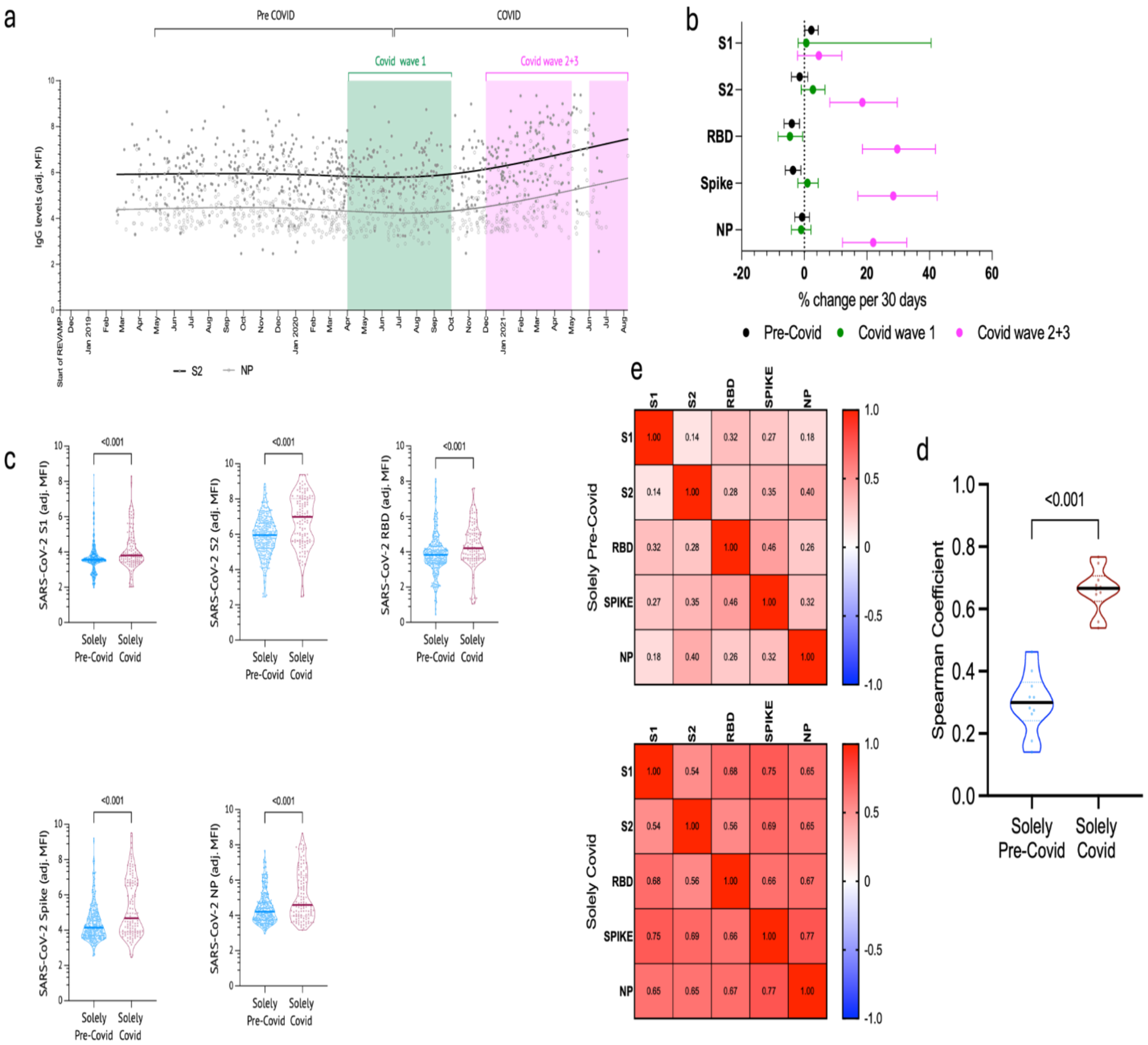
a
Pre COVID
COVID
Covid wave 1
Covid wave 2+3
IgG levels (adj. MFI)
Start of REVAMP
Jan 2019
Jan 2020
Jan 2021
S2
NP
b
S1
S2
RBD
Spike
NP
% change per 30 days
Pre-Covid
Covid wave 1
Covid wave 2+3
c
SARS-CoV-2 S1 (adj. MFI)
SARS-CoV-2 S2 (adj. MFI)
SARS-CoV-2 RBD (adj. MFI)
SARS-CoV-2 Spike (adj. MFI)
SARS-CoV-2 NP (adj. MFI)
<0.001
Solely Pre-Covid
Solely Covid
d
Spearman Coefficient
<0.001
Solely Pre-Covid
Solely Covid
e
Solely Pre-Covid
Solely Covid
S1
S2
RBD
SPIKE
NP
